# Supplementary material for: The prognosis of different distant metastases pattern in malignant tumors of the adrenal glands: A population-based retrospective study
Source: PLoS One. 2022 Mar 15;17(3):e0264431. doi: 10.1371/journal.pone.0264431 (PMC8923449; doi:10.1371/journal.pone.0264431)
Supplement: Attachment — Submitted filename: Response letter for PLOS ONE .doc [file pone.0264431.s001.doc]

Editorial Office,

PLOS ONE	September 3rd, 2021

Dear Prof. Filomena de Nigris,

Thank you for your letter and advice regarding the review and revision of our manuscript entitled “**The prognosis of different distant metastases pattern in malignant tumors of the adrenal glands: A population-based retrospective study**”. The submission ID is **PONE-D-21-11886.** We have addressed all of the comments raised by the reviewers and have revised the paper accordingly. The amendments are highlighted in the revised manuscript. A point-to-point reply is included as follows. We are grateful to the high appreciations and constructive suggestions on our manuscript from you and three reviewers.

We would like to re-submit the revised manuscript for your consideration. We hope that the revision is acceptable, and we look forward to hearing from you soon.

Best Regards!

Yours sincerely,

Haibin Wei MD, and Da-hong Zhang, M.D.

1. Haibin Wei, Department of Urology, Zhejiang Provincial People's Hospital, People's Hospital of Hangzhou Medical College, No. 158, Shangtang Road, Xiacheng District, Hangzhou 310014, Zhejiang, China. Tel. +86 137 7786 0207. Fax: +86 571 85131448. Email address: whb-sysu@163.com.

2. Dahong Zhang, Department of Urology, Zhejiang Provincial People's Hospital, People's Hospital of Hangzhou Medical College, No. 158, Shangtang Road, Xiacheng District, Hangzhou 310014, Zhejiang, China. Tel. +86 571 8589 3312. Fax: +86 571 85131448. ORCID: 0000-0002-6934-7956. Email address: zhangdahong88@yeah.net.

We would like to express our sincere gratitude to the reviewers for their constructive and positive comments.

**Reviewer #1:**

Dear Editor, Zhang the interesting paper by ..et al the aims to investigate the impact of different 24 distant metastases pattern on the survival of patients with adrenal malignancy.

**1.**	Major finds comes from the fact that many confounding factors are not evaluated such as tumor size, treatment regime, surgery, grade of tumor, so these events influence the follow up of patient and prognosis.

**Response:** The reviewer’s comment is well appreciated. Thank you for the reminder.

We do agree with you that some factors influence the follow up of patient and prognosis, such as tumor size, treatment regime, surgery, grade of tumor.

First, according to your comment, we added the variable “surgery” and defined the variable as the corresponding definition of the SEER database. As for table 2, we added “As for surgery, the distribution of patients was statistically significant except brain metastasis (P < 0.05).” to the corresponding section of the revised manuscript. As for univariate analysis, we added “And patients with surgery had better OS and CSS than those without (P < 0.001).”

Second, we have added new variables like tumor size and grade of tumor in table 1 and table 2 and made the corresponding changes in all relevant section of the manuscript. For table1, we added the corresponding description as “In addition to the grade of unknown, the most common grade was poor differentiated (30.9%).” and “48.3% of patients had tumor size ≤100mm, but 19.1% had unknown tumor size.” in the revised manuscript.

Third, although “SEER research data, 18 registries, Nov 2019 Sub (2000- 2017)” database has different variables and a larger sample size than other database, some of the data is incomplete, such as pathological grade. For the grade of tumor, the data for 82 % of the adrenal cortical carcinoma and 36.4% of the neuroblastoma were unknown, respectively. So we did not include it in univariate survival analysis to avoid the bias of incomplete data.

Fourth, this study focuses on evaluating prognosis of different distant metastases pattern in adrenal malignancy patients at the first diagnosis. We aimed to provide a better understanding of the prognosis before diverse treatments. Therefore, the other treatment strategies were not included in the study.

Thank you very much for your great comment.

**2.** Another major point is that in lane 169 the authors indicated that site of metastases was an independent prognostic factor (P <0.05) from the table 4 seams that brain metastasis were statistically associated with prognosis

**Response:** The reviewer’s comment is well appreciated. Thank you for your reminding. According to your comment, we have changed the sentence into “The parameters, including metastatic site, gender, histology, primary site, residential areas and surgery, were selected in multivariate analysis. As for metastatic site, brain metastasis was still the worst prognostic metastasis both in OS and CSS. The same as the univariate analysis, primary site was an independent prognostic factor for adrenal metastatic cancer patients both in OS and CSS (P < 0.05).” in the revised manuscript.

**3.** moreover in the presentation of data the authors should better indicate numerically if one condition improves the survival or prognosis.

**Response:** The reviewer’s comment is well appreciated. Thank you for your valuable advice.

First, we have added a new table titled “Frequencies of combination metastasis and 3, 5-y OS” in the revised manuscript. In this table, the 3, 5-y overall survival and median OS were shown numerically.

Second, in our study, with respect to the surgery, patients (suffering from a single metastasis) underwent the surgery showed the better prognosis for OS and CSS in univariate survival analysis and multivariable survival analysis. We do agree with you that specific digitization may be a good choice if some condition improves the survival or prognosis. This study focuses on prognosis of different distant metastases pattern, not on therapeutic intervention for metastatic adrenal malignancy patients. The optimization of intervention conditions will be an important part of subsequent research.

Thanks for your suggestion.

**4.** In tables are not indicated how many patients had one or more metastasis reported instead in Kaplan analysis.

**Response:** The reviewer’s comment is well appreciated. Thank you for your reminding. We have added a new table about the frequency of one or more metastasis.

First, the number of one site, two sites, three sites, and four sites are shown in Table 3, respectively.

Second, according to your comment, we have added “The metastatic pattern of metastatic adrenal malignancy was exhibited in Table 3. Theoretically there were 15 metastatic forms, including 4 single metastases and 11 combinations of metastases. However, there were no relevant cases in this study in two types of metastatic forms, which were brain and liver metastases and brain and liver and lung metastases. We found bone metastasis was the most common metastasis in single metastatic patients (24.08%), followed by liver (15.71%), lung (12.04%) and brain (0.71%). As for two sites, the highest frequency was observed in patients with liver and lung metastases at 10.51% (103/980). Lung metastasis presented better early survival rate in single metastasis. Patients with brain and lung metastases had the worse survival rate than other metastatic types in two sites metastases” in the revised manuscript.

Thank you for your suggestion.

**5.** Minor points

The Ven diagramm needs a color legend

The quality of image is poor

**Response:** The reviewer’s comment is well appreciated. We have modified the image according to journal’s requirements. We are grateful to the constructive suggestions on our manuscript from you.

**Reviewer #2:**

The main points that the authors should clarify are the following:

1. what is the percentage of metastatic cancer at the time of diagnosis.

**Response:** Thank you very much for your comment. According to your comment, we have added “A total of 2053 eligible patients with primary adrenal malignancy between 2010 and 2017 were identified from SEER database. Among these patients, the metastatic adrenal malignancy accounted for 47.74 %( 980/2053) at the time of diagnosis, including 493 men (50.3%) and 487 women (49.7%). The metastatic information on bone, brain, liver, and lung metastasis was collected in the SEER database." in the revised manuscript.

**2.** how many patients have more than one metastasis.

**Response:** The reviewer’s comment is appreciated. We added a new table about the frequency of four single metastases and 9 combinations of metastases. The number of one site, two sites, three sites, and four sites are shown in Table 3, respectively.

**3.** since it is not clear what the median survival range of subjects presenting metastases to bone versus brain is, the authors should better comment on the Kaplan-Meier analysis and indicate in the text which was the best survival with several metastatic sites

**Response:** Thank you for your reminding. We have added a new table about the median survival and the frequency of combination metastasis in this manuscript (Table 3). And we added “The metastatic pattern of metastatic adrenal malignancy was exhibited in Table 3. Theoretically there were 15 metastatic forms, including 4 single metastases and 11 combinations of metastases. However, there were no relevant cases in this study in two types of metastatic forms, which were brain and liver metastases and brain and liver and lung metastases. We found bone metastasis was the most common metastasis in single metastatic patients (24.08%), followed by liver (15.71%), lung (12.04%) and brain (0.71%). As for two sites, the highest frequency was observed in patients with liver and lung metastases at 10.51% (103/980). Lung metastasis presented better early survival rate in single metastasis Patients with brain and lung metastases had the worse survival rate than other metastatic types in two sites metastases. In addition, median OS cannot be concluded in only bone metastasis and bone and brain metastases in Kaplan-Meier analysis.” in the revised manuscript.

**4.** in the conclusions section, authors should more clearly describe whether knowledge of these differences in metastatic patterns could help better guide pre-treatment evaluation of cancer and determine interventions with curative intent.

**Response:**

The reviewer’s comment is well appreciated. Thank you for your constructive suggestion. We do agree with you that it is very necessary to clearly articulate the significance of metastatic patterns for pre-treatment evaluation and clinical interventions.

First, various prognostic values are associated with different metastatic sites, and knowledge of overall survival is very important for pre-treatment evaluation. According to your comment, we have added “The nomogram may help predict the 1-year, 3-year and 5-year overall survival in patients with primary metastatic adrenal malignancy.” of in the conclusions section of the revised manuscript.

Second, the phenomenon that metastatic neuroblastoma had better oncological survival than metastatic adrenal cortical carcinoma was observed in our study. Therefore, the prognosis of different pathological types is completely different for adrenal malignancy, which may remind us to choose different interventions with curative intent for different pathological types of tumors. According to your comment, we have changed this sentence to “Knowledge of these differences in metastatic patterns and pathological types may contribute to designing targeted pre-treatment assessment of adrenal malignancy and making a personalized curative intervention.” of in the conclusions section of the revised manuscript.

Third, diagnosis and treatment do not equate with equivalent benefit for metastatic adrenal malignancy, and further understanding of outcome of adrenal malignancy, especially metastatic adrenal malignancy, might help make reasonable medical decision and save the unnecessary expend on the advanced tumor. According to your comment, we have added this part in the introduction part due to the limited space in the conclusion part.

Fourth, we believe that pre-treatment evaluation and treatment decision-making is a gradual process, with the deepening of understanding of tumor biological behavior, development of new drugs and inspection methods, and the diversification of treatment methods. Therefore, we hope that subsequent research can help better guide pre-treatment evaluation of cancer and determine interventions with curative intent. According to your comment, we have added “Further efforts are still need to investigate the relationship between more comprehensive factors and adrenal malignancy in the future.” of in the conclusions section of the revised manuscript.

Thank you very much for your great comment.

**5.** Minor points

1) Authors should submit a figure legend for Figure 1 Figure 1

2) Authors should provide image with higher resolution, since image quality is poor

**Response:** The reviewer’s comment is well appreciated. We have added the figure legend in Figure1. Meanwhile, we have modified the image and upload the corresponding image. Thank you for your suggestion.

**Reviewer #3:**

Dear Editor

In the present study, Zhang.et al performed several experiments aimed to prove the impact of different 24 distant metastases pattern on the survival of patients with adrenal malignancy.

The work is well elaborated, and the focus is very interesting. However, I find some parts of the article need to be improved and I have some revisions to add before acceptance. I recommend acceptance after revision.

Major finds

**1.** The authors should improve the clinical data added the percentage of metastatic cancer at diagnosis.

**Response:** Thank you for your reminding. According to your comment, we have added “A total of 2053 eligible patients with primary adrenal malignancy between 2010 and 2017 were identified from SEER database. Among these patients, the metastatic adrenal malignancy accounted for 47.74 %( 980/2053) at the time of diagnosis, including 493 men (50.3%) and 487 women (49.7%). The metastatic information on bone, brain, liver, and lung metastasis was collected in the SEER database.” in the revised manuscript. Thank you for your suggestion.

**2.** The authors should clarify which is the mean survival of metastases with bone compare to brain

**Response:** The reviewer’s comment is appreciated. We have added a new table titled “Frequencies of combination metastasis and 3, 5-y OS” in the revised manuscript. In this table, the median survival time of all metastatic forms was presented. Brain metastasis showed worst survival outcomes in single metastasis. Thank you for your suggestion.

**3.** The author should improve the comment about the “Kaplan analysis” and highlight which was the best survival with different metastatic sites in the text

**Response:** The reviewer’s comment is well appreciated. We added the new table which clarified the survival rate and median survival. Meanwhile, we added “The metastatic pattern of metastatic adrenal malignancy was exhibited in Table 3. Theoretically there were 15 metastatic forms, including 4 single metastases and 11 combinations of metastases. However, there were no relevant cases in this study in two types of metastatic forms, which were brain and liver metastases and brain and liver and lung metastases. We found bone metastasis was the most common metastasis in single metastatic patients (24.08%), followed by liver (15.71%), lung (12.04%) and brain (0.71%). As for two sites, the highest frequency was observed in patients with liver and lung metastases at 10.51% (103/980). Lung metastasis presented better early survival rate in single metastasis. Patients with brain and lung metastases had the worse survival rate than other metastatic types in two sites metastases. In addition, median OS cannot be concluded in only bone metastasis and bone and brain metastases in Kaplan-Meier analysis.” in the revised manuscript.

Thank you so much for your comment.

**4.** In the "Conclusion" section the authors should better explain the impact of their work

**Response:** The reviewer’s comment is well appreciated. Thank you for your constructive suggestion. We do agree with you that it is very necessary to clearly articulate the significance of metastatic patterns for pre-treatment evaluation and clinical interventions.

First, various prognostic values are associated with different metastatic sites, and knowledge of overall survival is very important for pre-treatment evaluation. According to your comment, we have added “The nomogram may help predict the 1-year, 3-year and 5-year overall survival in patients with primary metastatic adrenal malignancy.” of in the conclusions section of the revised manuscript.

Second, the phenomenon that metastatic neuroblastoma had better oncological survival than metastatic adrenal cortical carcinoma was observed in our study. Therefore, the prognosis of different pathological types is completely different for adrenal malignancy, which may remind us to choose different interventions with curative intent for different pathological types of tumors. According to your comment, we have changed this sentence to “Knowledge of these differences in metastatic patterns and pathological types may contribute to designing targeted pre-treatment assessment of adrenal malignancy and making a personalized curative intervention.” of in the conclusions section of the revised manuscript.

Third, diagnosis and treatment do not equate with equivalent benefit for metastatic adrenal malignancy, and further understanding of outcome of adrenal malignancy, especially metastatic adrenal malignancy, might help make reasonable medical decision and save the unnecessary expend on the advanced tumor. According to your comment, we have added this part in the introduction part due to the limited space in the conclusion part.

Fourth, we believe that pre-treatment evaluation and treatment decision-making is a gradual process, with the deepening of understanding of tumor biological behavior, development of new drugs and inspection methods, and the diversification of treatment methods. Therefore, we hope that subsequent research can help better guide pre-treatment evaluation of cancer and determine interventions with curative intent. According to your comment, we have added “Further efforts are still need to investigate the relationship between more comprehensive factors and adrenal malignancy in the future.” of in the conclusions section of the revised manuscript.

Thank you very much for your great comment.

**5.** Minor points

The authors should improve the quality of images

The “Ven diagram” needs a color legend

**Response:** The reviewer’s comment is well appreciated. We have added the color legend in Figure1. Meanwhile, we have modified the image and upload the corresponding image. We are grateful to the constructive suggestions on our manuscript from you.
