# Supplementary material for: The prognosis of different distant metastases pattern in malignant tumors of the adrenal glands: A population-based retrospective study
Source: PLoS One. 2022 Mar 15;17(3):e0264431. doi: 10.1371/journal.pone.0264431 (PMC8923449; doi:10.1371/journal.pone.0264431)
Supplement: Attachment — Submitted filename: Response letter for PLOS ONE.doc [file pone.0264431.s002.doc]

Editorial Office,

PLOS ONE	December 4th, 2021

Dear Prof. Filomena de Nigris,

Thank you for your letter and advice regarding the review and revision of our manuscript entitled “**The prognosis of different distant metastases pattern in malignant tumors of the adrenal glands: A population-based retrospective study**”. The submission ID is **PONE-D-21-11886.** We have addressed all of the comments raised by the reviewers and have revised the paper accordingly. The amendments are highlighted in the revised manuscript. A point-to-point reply is included as follows. We are grateful to the high appreciations and constructive suggestions on our manuscript from you and three reviewers.

We would like to re-submit the revised manuscript for your consideration. We hope that the revision is acceptable, and we look forward to hearing from you soon.

Best Regards!

Yours sincerely,

Haibin Wei MD, and Da-hong Zhang, M.D.

1. Haibin Wei, Department of Urology, Zhejiang Provincial People's Hospital, People's Hospital of Hangzhou Medical College, No. 158, Shangtang Road, Xiacheng District, Hangzhou 310014, Zhejiang, China. Tel. +86 137 7786 0207. Fax: +86 571 85131448. Email address: whb-sysu@163.com.

2. Dahong Zhang, Department of Urology, Zhejiang Provincial People's Hospital, People's Hospital of Hangzhou Medical College, No. 158, Shangtang Road, Xiacheng District, Hangzhou 310014, Zhejiang, China. Tel. +86 571 8589 3312. Fax: +86 571 85131448. ORCID: 0000-0002-6934-7956. Email address: zhangdahong88@yeah.net.

We would like to express our sincere gratitude to the reviewers for their constructive and positive comments.

**Reviewer #4:**

The authors have greatly improved their manuscript by the recent revisions. My suggestions are very minimal, and are made mostly to increase the rigor and reproducibility of the manuscript.

**1.**	For all listed p-values (both in the text and in the tables), please provide the hypothesis test method that was used to obtain those values.

**Response:** The reviewer’s comment is well appreciated. Thank you for the reminder.

First, we used chi- Square test in table2. Depending on the conditions of use, the p-values we obtained were derived from Pearson's chi-square test as well as Fisher exact test. Pearson’s chi-square test and Fisher’s exact test are tests of categorical variables. Pearson’s chi-square test assumes that the data: 1) are a simple random sample, 2) are of sufficient sample size and 3) are independent of each other. When cells are sparsely populated, considered any cell value is less than 5 with the traditional rules, Fisher’s exact test was employed [PMID: 28295394]. To be more precise, we have added corresponding annotation of different types of statistical methods in table2.

Second, COX regression model was used in table4 and table5. COX regression models can well address the diversity of survival time distributions in survival data, and can better perform censored data. We have used univariate COX regression analysis in table4 and multivariate COX regression analysis in table5.

Third, Kaplan-Meier method is often applied to estimate the probability of survival, and it was used in figure2 and figure3. We analyzed the data with log-rank test, which was considered an appropriate test for survival analysis [PMID: 28962743].

Thank you very much for your great comment.

**2.** For age, it appears that data is reported as median +/- IQR, which is appropriate if the data is not normally distributed or skewed. However, it appears the hypothesis test method used on the age data is a student's t-test which has the assumption that that data is normally distrubted. This is a discrepancy. The authors should check whether the age data is normally distributed. If so, a t-test is appropriate and mean +/- SD should be reported. If the data is not normally distributed, then a Mann Whitney test should be used and median +/- IQR should be reported.

**Response:** The reviewer’s comment is well appreciated. Thank you for your reminding.

First, we have checked that the age data is not normally distributed. So we used Mann Whitney test and reported median. You can find the median +/- IQR in Table1.

Second, we also checked the age data was not normally distributed in table 2 and added median +/- IQR for more completeness.

Third, we reviewed our manuscript under your instruction and found the mistake in Method section. For more rigorous, we revised the sentence into “Categorical variables were compared using chisquare tests, and continuous variables were analyzed by the Mann Whitney test for non-normal distribution” in the article.

Thank you for your suggestion.

**3.** There appear to be some variables that have very small counts (less than 5). It is unclear why a chi-square test was used to analyze these instead of the more appropriate fisher exact test. Please rectify or indicate why the chisquare test is appropriate for all categorical variables, even when the sample size is small.

**Response:** The reviewer’s comment is well valuable. Thank you for your suggestion.

First, as your comment, fisher exact test were more appropriate for use when one or more cells has a count of less than 5. We have conducted fisher exfact act for those variables actually. For table2, we selected appropriate P value for different variables according to the different conditions. To better illustrate the statistical method from which the P values are taken, we have added the corresponding P-values annotations as a and b in table 2. As for annotation a, the P value was selected from Pearson chi-Square.

Second, we believe that we have been misrepresented our statistical method in this paragraph. In SPSS 25, chi-Square included Pearson chi-Square, likelihood ratio and fisher exact test. We were going to describe chi-Square was used to analyze rather than Pearson chi-Square. To be more precise, we have changed the sentence into “Categorical variables were compared using chisquare tests, and continuous variables were analyzed by the Mann Whitney test for non-normal distribution.” in the article.

Third, take variable primary site for example, the P value was selected from Pearson chi-Square when comparing the patients with bone metastasis and without bone metastasis. As for brain metastasis, the P value was selected from fisher exact test, as shown in the figure below.

Thank you for your reminder.

**4.** For Table 2, please indicate what hypothesis test was used to obtain each p-value.

**Response:** The reviewer’s comment is well appreciated. Thank you for your reminding.

In table 2, we aimed to verify that there are differences in patients with metastasis and without metastasis for different variables. We used chi-Square test in table2. Depending on the conditions of use, the p-values we obtained were derived from Pearson's chi-square test as well as Fisher exact test. As shown in QUESTION1.

Take variable gender for example.

First, establishing test hypotheses and determining test levels.H0: the males and females have the same probability of suffering adrenal malignancy with bone metastasis and without bone metastasis,π1=π2.H1: the males and females have different probability of suffering adrenal malignancy with bone metastasis and without bone metastasis, π1≠π2 α=0.05.

Second, calculate the test statistic and obtain the p-value. In this hypothesis test, every cell is more than 5, Pearson Chi-square is reasonable accurate. And we can acquire the P value < 0.001, as shown the figure below.

Third, At the level of α =0.05, reject H0,accept H1, the distribution of gender among the patients with bone metastasis and without bone metastasis was significantly different.

Thank you for your suggestion.

**5.** For Table 3, what is the difference between NA & /? Why is median OS unable to be calculated for 2 or more situations?

**Response:** The reviewer’s comment is well appreciated.

First, / was used to indicate that the median OS cannot be calculated due to the absence of related cases. The number of cases of brain and liver metastases and brain and liver and lung metastases were zero. Therefore median OS cannot be calculated

Second, NA was used to present that the median OS unable to display even though the number of related cases were sufficient. The median OS is defined as “the length of time from either the date of diagnosis or the start of treatment for a disease, such as cancer, that half of the patients in a group of patients diagnosed with the disease are still alive” in NCI's Dictionary, as shown in the figure below.

The median OS of those two group cannot be concluded because more than half of the patients still alive at the end of the follow up. Take the bone metastasis for example, we redrawn the survival curve, as shown in the figure below.

In this figure, we added the survival median line, and the curve of bone metastasis are consistent on the median line. So the median OS cannot be calculated in only bone metastasis and bone and brain metastases.

Third，to better present the difference between the NA & /, we have added corresponding annotations below the table. The annotation showed “the median OS cannot be concluded because more than half of the patients still alive at the end of the follow up” for NA and “the median OS cannot be calculated due to the absence of related cases” for /.

Thank you for your suggestion.

6. For the Cox Proportional Hazards models, it would be nice to know why/how the variables were chosen to be included/excluded from the modeling. For instance, there are far more variables reported in Table 1 than used in the Cox modeling. It is unclear why this is so. Please justify.

**Response:** Thank you very much for your comment.

This is a very interesting question. We did not present all variables reported in Table 1 but we have verified and included the variables with statistically significant. The variable such as *years of diagnosis, laterality, median income* were excluded because the *p* >0.05 and we commonly assumed that these variables are not significantly different. Other variables such as *grade* were excluded because the category *unknown* was unreasonably numerous. So we excluded variables that have no significance in the Cox modeling in order to be more concise and precise.

Thank you for your question.

We are grateful to the constructive suggestions on our manuscript from you.
